# SSA with CWT and *k*-Means for Eye-Blink Artifact Removal from Single-Channel EEG Signals

**DOI:** 10.3390/s22030931

**Published:** 2022-01-25

**Authors:** Ajay Kumar Maddirala, Kalyana C. Veluvolu

**Affiliations:** School of Electronics Engineering, College of IT Engineering, Kyungpook National University, Daegu 41566, Korea; maddirala@knu.ac.kr

**Keywords:** electroencephalogram (EEG), electrooculogram (EOG), singular spectrum analysis (SSA), continuous wavelet transform (CWT), *k*-means clustering

## Abstract

Recently, the use of portable electroencephalogram (EEG) devices to record brain signals in both health care monitoring and in other applications, such as fatigue detection in drivers, has been increased due to its low cost and ease of use. However, the measured EEG signals always mix with the electrooculogram (EOG), which are results due to eyelid blinking or eye movements. The eye-blinking/movement is an uncontrollable activity that results in a high-amplitude slow-time varying component that is mixed in the measured EEG signal. The presence of these artifacts misled our understanding of the underlying brain state. As the portable EEG devices comprise few EEG channels or sometimes a single EEG channel, classical artifact removal techniques such as blind source separation methods cannot be used to remove these artifacts from a single-channel EEG signal. Hence, there is a demand for the development of new single-channel-based artifact removal techniques. Singular spectrum analysis (SSA) has been widely used as a single-channel-based eye-blink artifact removal technique. However, while removing the artifact, the low-frequency components from the non-artifact region of the EEG signal are also removed by SSA. To preserve these low-frequency components, in this paper, we have proposed a new methodology by integrating the SSA with continuous wavelet transform (CWT) and the *k*-means clustering algorithm that removes the eye-blink artifact from the single-channel EEG signals without altering the low frequencies of the EEG signal. The proposed method is evaluated on both synthetic and real EEG signals. The results also show the superiority of the proposed method over the existing methods.

## 1. Introduction

Electroencephalogram (EEG) signals represent the electrical activity of the brain and are measured by placing electrodes over the scalp. The EEG signals are often used to understand brain functions such as mental state (or cognitive state) and brain disorders such as epilepsy and stroke [1,2,3,4,5]. However, the recorded EEG signals are always contaminated by physiological artifacts, such as electrooculogram (EOG), electromyogram (EMG) and electrocardiogram (ECG). Unlike other artifacts, the EOG artifact that is a result of the eye-blink/movement activity and always contaminates the EEG signal. As the eye-blink is an uncontrollable and involuntary activity and occurs once every 5 s (as in [6]), we refer to the EOG artifact as an eye-blink artifact in this paper. Therefore, the removal of these artifacts forms an important stage before analyzing the EEG signals [6]. Hence, methods such as linear filters have been used for eye-blink artifact removal from EEG signals. In general, the eye-blink artifact strongly contaminates the low-frequency spectrum of EEG (0.5–12 Hz) [7]. Therefore, the use of linear filters for the removal of eye-blink artifacts alters the valuable information from the EEG signal. Later, a regression-based method was proposed to remove artifacts from multichannel EEG signals [8]. In this method, the artifact weighting coefficients are computed from the EOG channels, which are recorded separately. However, such fixed coefficients may not fully remove the eye-blink artifacts from the EEG signals.

Blind source separation (BSS) techniques such as independent component analysis (ICA) and canonical correlation analysis (CCA) techniques have been used to remove artifacts from the multichannel EEG signals [9,10,11,12,13]. The ICA technique was extensively used to remove eye-blink artifacts from EEG signals as compared to the CCA method [12,13]. Several other techniques were also integrated with ICA for efficient removal of eye-blink artifacts from the multichannel EEG signals [14,15,16,17]. The artifact subspace reconstruction (ASR) method was also proposed to remove the artifact from the EEG signals [18,19]. The performance of this method depends on the user-defined cut-off parameter *k*. Even though a detailed study was conducted for selecting the cut-off parameter in [19], inappropriate selection of this parameter may result in the loss of EEG information.

Recently, the demand for in-home health monitoring has been increasing due to the increase in chronic illnesses and population aging [20]. Several studies have employed portable EEG devices for various applications, including analysis of cognitive state in stroke survivors, sleep disorders, driver fatigue and event-related potential (ERP)-based BCI applications [2,21,22,23]. To reduce the burden and to minimize the stress on the patient, recently portable EEG devices with a reduced number of EEG channels, including single EEG channel equipment [24,25], have been developed. Therefore, the existing ICA and ASR techniques that are popular for multichannel settings cannot be used to remove eye-blink artifacts from single-channel EEG signals. Therefore, there is a need for new methods that are customized for processing single-channel EEG signals.

An adaptive filter is one of the possible solutions to process single-channel EEG signals. The use of adaptive filters to remove eye-blink artifacts from the EEG signals was first discussed in [26]. However, the adaptive filters require reference signals to remove the eye-blink artifacts from single-channel EEG data. Therefore, in [27], the adaptive filter is combined with discrete wavelet transform (DWT) to solve this problem. In this method, the reference signal (an approximated eye-blink artifact) needed for the adaptive filter is estimated from the contaminated EEG signal using DWT. After that, the estimated eye-blink artifact signal is used as a reference signal to the adaptive filter to remove the eye-blink artifact from the EEG signal. Recently, the Savitzky–Golay (SG) filter was also used to estimate the reference signal needed for an adaptive filter [28]. Very recently, the Variational Mode Extraction (VME) and DWT techniques were combined to remove eye-blink artifacts from single-channel EEG signals [29]. In this method, first, the eye-blink artifact interval is identified using VME. Next, a DWT algorithm is employed to filter the contaminated interval of the EEG signal. Although this method does not significantly alter the non-artifact regions of the EEG signal, the eye-blink artifact component is partially removed from the contaminated EEG signal. Along with these methods, a data-driven decomposition method, namely an ensemble empirical mode decomposition with adaptive noise, is also proposed to remove eye-blink artifacts from a single-channel EEG signal [30]. However, this method alters the non-artifact regions of the EEG signal.

Singular spectrum analysis (SSA) is a subspace-based technique used to extract the low-frequency, oscillating and noise components from uni-variate time-series data [31,32]. Recently, the SSA technique has been applied for processing the biomedical signals [33,34,35,36]. The application of SSA for eye-blink artifact removal from single-channel EEG signals was first studied in [37]. However, identifying the desired signal subspace (eigenvectors) is a critical step in classical SSA. Therefore, new criteria were proposed in [38] to identify the eigenvectors that are used to reconstruct the desired signal. In [38], the SSA is combined with an adaptive filter to enhance the performance of the adaptive filter over the method in [27]. Recently, in [39], with new grouping criteria, the adaptive SSA technique is combined with ANC (SSA+ANC) and the method showed better performance over the method in [38]. Moreover, SSA is used as a means to apply ICA on single-channel EEG signals [40,41]. Very recently, SSA has been used as a smoothing filter in [42] to remove the eye-blink artifact from the EEG signal. In this method, the user has to adjust the threshold for faithful separation of the eye-blink artifact from the EEG signal. In other words, the performance of the method is sensitive to the user-defined threshold.

Even though the SSA is able to extract the eye-blink artifact efficiently, it also removes the EEG low-frequency information (0.5–12 Hz) from the non-artifact regions. Removing these components may affect the subsequent analysis of the EEG signal. Recently, the effect of pre-processing methods on EEG results has been studied in [43] and it concludes that the selection of artifact removal strategy affects the end application results. Therefore, care should be taken while designing the artifact removal method. Therefore, in this paper, we proposed a new technique by combining SSA with continuous wavelet transform (CWT) and *k*-means algorithms so that it removes the eye-blink artifact from single-channel EEG signal without altering the non-artifact regions of the EEG signal. The proposed method exploited the strengths of both SSA and the CWT in removing the artifact. Unlike the method in [42], where time-domain features are used, the proposed method used frequency-domain features of the signal to remove the eye-blink artifact. Moreover, a frequency-based threshold is defined for SSA to identify the artifact subspace, and such threshold will act as the cut-off frequency as in a low-pass filter. The performance of the proposed method (which we call SSA-CWT) is evaluated on synthetic and real single-channel EEG signals. The results show its superiority over existing methods.

The rest of the paper is organized as follows: The performance measures to evaluate the efficiency of the proposed and existing method are defined in Section 2. The framework of the proposed method is discussed in Section 3. The simulation results and their discussions are presented in Section 4 and Section 5, respectively. Section 6 concludes the paper.

## 2. Performance Metrics

In this section, we have employed several few performance metrics to evaluate the performance of the proposed method on a synthetic EEG dataset. We define four commonly used performance measures to evaluate the performance of the methods on synthetic EEG data: the relative root mean square error (RRMSE), the canonical correlation analysis (CC), artifact reduction ratio (λ) and mean absolute error (MAE). To evaluate the performance of the proposed method on real EEG datasets, we first identify the non-artifact and artifact intervals of the real EEG signal manually. Then RRMSE and CC between the non-artifact interval of contaminated and corrected EEG signals is computed.

Consider the *N* sampled contaminated signal x=s+p.a, where s and a are the true EEG and the EOG artifact signals, respectively and *p* is an artifact mixing constant. The following performance metrics are defined as follows:

### 2.1. Relative Root Measure Square Error (RRMSE)

The RRMSE measure is often used to evaluate the performance of artifact removal methods on synthetic EEG data. The RRMSE between the two signals a and $\hat{a}$ can be defined as

(1) $$RRMSE=\sqrt{\frac{\sum_{n=1}^{N}{\left[a\left(n\right)-\hat{a}\left(n\right)\right]}^{2}}{\sum_{n=1}^{N}a^{2}\left(n\right)}}\times 100\left(\%\right)$$

where a and $\hat{a}$ represent the ground truth eye-blink and the estimated eye-blink artifacts, respectively. The relationship between the signal-to-noise ratio (SNR) and the artifact mixing constant *p* is given by

$$SNR=\frac{RMS(s)}{RMS\left(pa\right)}$$

$$RMS\left(s\right)=\sqrt{\frac{1}{N}\sum_{n=1}^{N}s^{2}\left(n\right)}$$

when the constant *p* is small, the EOG artifact is small and the SNR of the EEG signal is high. The low RRMSE value indicates a good estimation of artifacts by the method. Here, the RRMSE is computed between the true eye-blink and the estimated eye-blink artifact to understand the efficacy of the proposed method in estimating the artifact from the contaminated EEG signal.

### 2.2. Correlation Coefficient (CC)

It is a statistical-based measure, which shows the strong relationship between the two signals. The CC measure is also used to evaluate the performance of an artifact removal technique. The CC between the two signals a and $\hat{a}$ can be defined as

(2) $$\mathrm{CC}=\frac{cov(a,\hat{a})}{\sigma_{aa}\sigma_{\hat{a}\hat{a}}}$$

where $cov\left(\cdot \right)$ represents the covariance between the two signals a and $\hat{a}$ and $\sigma_{\left(\cdot \right)}$ variance of the signal itself. The CC value close to one indicates a good estimation of eye-blink artifact from the contaminated EEG data.

### 2.3. Artifact Reduction Ratio (λ)

Along with the above-defined two performance measures, we also employed a performance metric that quantifies the percentage reduction in artifacts and is defined as

(3) $$\lambda =\left(1-\frac{R_{clean}-R_{after}}{R_{clean}-R_{before}}\right)\times 100$$

where $R_{clean}$ is set to 1 and the $R_{before}$ is the correlation between the true EEG and the contaminated EEG signals and $R_{after}$ is the correlation between the true EEG and the estimated EEG signals. For a good artifact removal method, this value should be high.

### 2.4. Mean Absolute Error (MAE)

This metric is employed to evaluate the performance of the proposed method in the frequency domain. It is defined as the sum of the absolute of the difference between the true EEG signal power spectrum, $P_{s}$, and the corrected EEG signal power spectrum $P_{\hat{s}}$ in a particular band. The MAE between the spectrums of the true and corrected EEG signals is defined as

(4) $$MAE=\frac{\sum_{i=1}^{K}\left|P_{s}\left(i\right)-P_{\hat{s}}\left(i\right)\right|}{K}$$

where *K* represents the number of frequency bins in a specific band. The MAE value is expected to be very small for a good artifact removal method.

### 2.5. Precision and Accuracy

Along with these performance measures, we have also defined two measures associated to binary classification, precision and accuracy, to detect how precisely and accurately the proposed method identifies (detected) the artifact and non-artifact intervals of the EEG signals. The performance measures, precision and accuracy are defined as

(5) $$\mathrm{Precision}=\frac{TP}{\left(TP+FP\right)}$$

(6) $$\mathrm{Accuracy}=\frac{TP+TN}{\left(TP+TN+FP+FN\right)}$$

where TP,TN,FP and FN are true positive, true negative, false positive and false negative, respectively. The true positive indicates that the artifact removal method correctly predicted (detected) the positive class (artifact interval) and true negative indicates that the method correctly detected the negative class (non-artifact interval). Similarly, false positive and false negatives represents that the method incorrectly detected the positive and negative classes, respectively.

## 3. Eye-Blink Artifact Removal from Single-Channel EEG Signals

**Figure 1:**
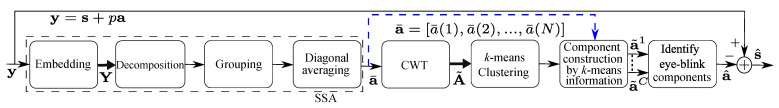
Block diagram of proposed method for eye-blink artifact removal from single EEG signals.

The key components of the proposed method for eye-blink artifact removal is shown in Figure 1. It is a two-step approach: first, an eye-blink artifact is extracted from the contaminated single-channel EEG signal using SSA. Next, the extracted eye-blink artifact is denoised in the non-artifact region using CWT and k− means algorithms.

SSA is a data-driven technique employed to process the single-channel (uni-variate) time-series data [31,32]. Basically, the SSA technique comprises the following four steps: embedding, decomposition, grouping and diagonal averaging. Let us consider the contaminated EEG signal y, which is a result of the mixing model shown as follows:

(7) y=s+pa

where s and a are the ground truth EEG and the eye-blink artifact signals, respectively, and *p* is an artifact mixing constant that changes the signal-to-noise ratio (SNR) of the measured EEG signal y. When *p* is small (<1), the artifact contribution is less and results in a high SNR of the EEG signal y and vice-versa for p>1. The key steps of SSA are as follows: in the embedding step of SSA, the given *N* sampled single-channel EEG signal y=[y(1),y(2),…,y(N)] is mapped into multivariate data matrix Y.

(8) $$Y=\left[\begin{array}{ccccc} y(1) & y(2) & \ldots \ldots & \ldots \ldots & y(K)\\ y(2) & y(3) & \ldots \ldots & \ldots \ldots & y(K+1)\\ \vdots & \vdots & \ldots \ldots & \ldots \ldots & \vdots \\ y(M) & y(M+1) & \ldots \ldots & \ldots \ldots & y(N) \end{array}\right]$$

where *M* represents the window length and K=N−M+1. The matrix in (8) is called the Hankel matrix, as its anti-diagonal elements are constant (same). From (7), we can write Y=S+A (assuming that p=1), where S and A represent the trajectory matrices of the ground truth EEG and eye-blink artifact signals, respectively. Note that we have considered the artifact mixing constant p=1 for a simple explanation.

In the decomposition step of SSA, the trajectory matrix Y is decomposed into *M* trajectory matrices, for example, $Y_{1},Y_{2},\ldots ,Y_{M}$. Hence, the singular value decomposition (SVD) of $Y={\mathrm{UDV}}^{T}$ will be performed, where D represents the diagonal matrix whose elements are singular values and U and V are left and right singular matrices, whose columns are the eigenvectors of covariance matrix $C={\mathrm{YY}}^{T}$ and $C=Y^{T}Y$, respectively. However, direct decomposition of Y using SVD will increase the computational complexity. Therefore, the eigen decomposition of the covariance matrix of $C={\mathrm{YY}}^{T}$ will be performed first.

Let us consider that $\lambda_{1},\lambda_{2},\ldots ,\lambda_{M}$ and $u_{1},u_{2},\ldots ,u_{M}$ represent the eigenvalues and the eigenvectors of the covariance matrix $C={\mathrm{YY}}^{T}$. Moreover, we assume that the eigenvalues are sorted in the descending order of their amplitudes, $\lambda_{1}\geq \lambda_{2}\geq ,\ldots ,\geq \lambda_{M}\geq 0$. Then, the *j*th trajectory matrix $Y_{j}$ can be represented as

(9) $$Y_{j}=\sqrt{\lambda_{j}}u_{j}v_{j}^{T}\;j=1,2,\ldots ,M$$

from the SVD of Y, $v_{j}=Y^{T}u_{j}/\sqrt{\lambda_{j}}$. Substituting $v_{j}$ in (9), then the *j*th trajectory matrix $Y_{j}$ can be represented as

(10) $$Y_{j}=u_{j}u_{j}^{T}Y$$

The terms $u_{j}u_{j}^{T}$ in (10) form a subspace to reconstruct the *j*th component from the given signal y.

The main goal in the grouping step of SSA is to construct the eye-blink associated trajectory matrix A from *M* trajectory matrices $Y_{j},j=1,2,\ldots ,M$. Basically, we try to identify the appropriate eigenvectors by which we can construct an eye-blink artifact-associated trajectory matrix A. In the classical SSA technique, the eigenvectors that are used to construct the eye-blink artifact are identified based on the strength of the eigenvalues (eigen spectrum) of a covariance matrix C [36]. However, in this work, we have identified these eigenvectors based on the local mobility or Hjorth mobility [44], which is a signal complexity measure of each eigenvector [38]. Here, the hypothesis is that the local mobility of the eigenvectors corresponding to the eye-blink artifact is low and is high for eigenvectors associated with EEG signals. Therefore, the pre-defined threshold has to be set to identify the eigenvectors associated to the eye-blink artifact. In fact, finding the eigenvectors associated with the artifact is similar to identifying the artifact signal subspace from the given signal space. The parameter for identifying the eye-blink artifact subspace is computed as follows: as the eigenvector holds the variation of the data, first, *M* sampled sinusoidal signal of frequency *f* is generated. Next, the local mobility of the sinusoidal signal is computed and it will be used as a threshold. As the threshold, which is used to identify the artifact subspace, is proportional to the frequency, it is denoted with variable *f*. The threshold parameter *f* of SSA will be acting as a cut-off frequency as in the case of a low-pass filter. After identifying the eigenvectors (basis functions) associated with an eye-blink artifact, the trajectory matrix corresponding to an eye-blink artifact ($\bar{A}$) is computed using (10).

In fact, the computed trajectory matrix $\bar{A}$ that resulted from the grouping step of SSA will not hold the Hankel structure. In the diagonal averaging step of SSA, the anti-diagonal elements are replaced with their average, and the uni-variate signal $\bar{a}$ will be constructed using (11), as follows:

(11) $$\bar{a}\left(n\right)=\left\{\begin{array}{cc} \frac{1}{n}\sum_{i=1}^{n}\overset{-}{A}\left(i,n-i+1\right) & \mathrm{for}\;1\geq n<M\\ \frac{1}{M}\sum_{i=1}^{M}\overset{-}{A}\left(i,n-i+1\right) & \mathrm{for}\;M\geq n\leq K\\ \frac{1}{N-n+1}\sum_{i=n-K+1}^{N-K+1}\overset{-}{A}\left(i,n-i+1\right) & \mathrm{for}\;K<n\leq N \end{array}\right.$$

The extracted eye-blink artifacts $\bar{a}$ from the SSA method contain low-frequency EEG components. The direct subtraction of the extracted eye-blink artifact ($\bar{a}$) from the contaminated signal y results in a loss of low-frequency components in the reconstructed EEG signal. Therefore, denoising of these components from $\bar{a}$ has to be performed before it is subtracted from the contaminated EEG signal y.

### Denoising the EEG Components from the Extracted Eye-Blink Artifact ($\bar{a}$)

In order to denoise the EEG components in the extracted eye-blink artifact $\bar{a}$, we proposed a new methodology. In this method, the time-frequency representation of $\bar{a}$, which is the output of the SSA block, is performed using CWT, and it results in a matrix $\tilde{A}$ of size L×N and is denoted by

$$\left|\tilde{A}\right|=\left[\begin{array}{ccccc} \tilde{a}\left(1,1\right) & \ldots \ldots & \tilde{a}\left(1,j\right) & \ldots \ldots & \tilde{a}\left(1,N\right)\\ \tilde{a}\left(2,1\right) & \ldots \ldots & \tilde{a}\left(2,j\right) & \ldots \ldots & \tilde{a}\left(2,N\right)\\ \vdots & \vdots & \ldots \ldots & \ldots \ldots & \vdots \\ \tilde{a}\left(L,1\right) & \ldots \ldots & \tilde{a}\left(L,j\right) & \ldots \ldots & \tilde{a}\left(L,N\right) \end{array}\right]=\left[{\tilde{a}}_{1},\ldots ,{\tilde{a}}_{j},\ldots ,{\tilde{a}}_{N}\right]$$

where *L* is the number of frequencies for which CWT is computed. Each column vector ${\tilde{a}}_{j}$(j=1,2,…,N) of $\left|\tilde{A}\right|$ represents the feature vector of *j*th sample of $\bar{a}$. Next, each column vector of $\left|\tilde{A}\right|$ is clustered using *k*-means clustering algorithm with *C* number of clusters. Then, *k*-means algorithm provides the labels for each feature vector of $\left|\tilde{A}\right|$. These labels inform to which cluster a particular feature vector (indirectly the sample of $\bar{a}$) has fallen. With this clustering information, we construct *C* number of signals using (12)

(12) $${\tilde{a}}^{i}\left(j\right)=\left\{\begin{array}{cc} \bar{a}\left(j\right) & \mathrm{if}\;{\tilde{a}}_{j}\in C_{i},\;i=1,2,\ldots ,C\;\&\;j=1,2,\ldots ,N\\ 0 & \mathrm{if}\;{\tilde{a}}_{j}\notin C_{i} \end{array}\right.$$

Here, ${\tilde{a}}_{j}$ represents the *j*th column vector of matrix $\left|\tilde{A}\right|$. After decomposing the signal $\bar{a}$ into *C* number of signals, say ${\tilde{a}}^{1}$, ${\tilde{a}}^{2}$, ……, ${\tilde{a}}^{C}$ using (12), then, the fractal dimension (FD) [45] of each component is computed to identify the eye-blink artifact associated component. The estimated eye-blink artifact ($\hat{a}$) is identified based on the FD; usually, it is low for denoised eye-blink artifacts. Finally, the corrected EEG signal ($\hat{s}$) is obtained by subtracting the estimated eye-blink artifact $\hat{a}$ from y.

## 4. Results

To evaluate the performance of the proposed and the existing methods, we have constructed synthetically contaminated EEG signals from fatigue EEG data [46,47].

### 4.1. Construction of Synthetically Contaminated EEG Signal and Eye-Blink Artifact

**Figure 2:**
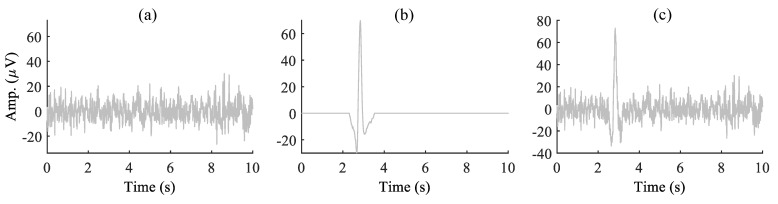
(**a**,**b**) Synthetically constructed ground truth EEG signal (s) and the EOG artifact (a), respectively, and (**c**) the contaminated EEG signal x=s+pa for the artifact mixing constant *p* = 0.5.

We have considered 10 subjects’ EEG data from the Fatigue EEG database [46,47]. Each subject performed a driving task on a static simulator. The EEG data were recorded in two phases normal and fatigue states using a 32-channel electrode cap with a sampling frequency of 1000 Hz. More details about the EEG data are discussed in [46,47]. In the construction of a true EEG signal for the simulation study, first, the raw EEG data measured from $Fp_{1}$ channel of ten subjects is down sampled to 250 Hz from 1000 Hz. Next, the baseline drift and the high-frequency components in the EEG data are removed using a band-pass filter with cut-off frequencies of 1 and 45 Hz. However, for synthetic simulation, a 10 s artifact-free EEG epoch is segmented from the filtered EEG data. These artifact-free EEG epochs are served as true EEG signals (s) for a synthetic simulation study. The synthetic eye-blink artifact data were constructed as follows: first, we identified the eye-blink artifact region manually and segmented it from the EEG signal. Next, zeros were padded to the segmented eye-blink component on both sides such that the length of the signal is 10 s. In order to remove the EEG remnants present on the eye-blink component, MATLAB smooth command was used. This results in the ground truth eye-blink artifact signal (a). We have constructed five such eye-blink artifacts from five subjects. Using these five eye-blink artifacts and ten EEG signals, we constructed a total of 50 synthetically contaminated EEG signals (y). However, we assumed that the contaminated EEG signal is additive mixing of both the true EEG signal and the eye-blink artifact, i.e., y=s+pa. Here, the artifact mixing constant *p* changes the SNR of the EEG signal. When the artifact mixing constant is p>1, the eye-blink artifact contribution in the contaminated EEG signal is high, and as a result, the SNR of the EEG signal is low. When p<1, the eye-blink artifact contribution in the contaminated EEG signal is low, and as a result, the SNR of the EEG signal is high. Figure 2 shows the synthetically constructed ground truth EEG, the eye-blink artifact and the contaminated EEG signals for p=0.5.

### 4.2. Parameter Settings for Proposed and Existing Methods

**Figure 3:**
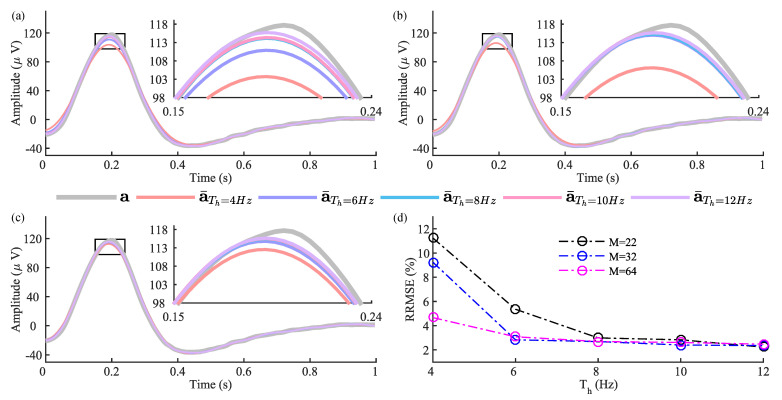
The estimated eye-blink artifacts $\bar{a}$ (the artifact region only) by SSA with different thresholds and the window lengths (**a**) M=22, (**b**) M=32, and (**c**) M=64. (**d**) Performance of SSA in terms of RRMSE for varying window length *M* and thresholds (f=4,6,8,10 and 12 Hz). The RRMSEs were calculated with respect to the ground truth eye-blink signal, a.

The faithful reconstruction of eye-blink artifact components from the contaminated EEG signal depends on the SSA window length and the parameter *f* that identifies the artifact subspace. Therefore, we have performed simulations to select these parameters. Figure 3 shows the effect of the parameter *f* and the window length *M* in extracting the eye-blink artifact. We have identified the region of the eye-blink component (the artifact region only) from the fifty extracted eye-blink artifact signals by SSA and computed the mean eye-blink artifact component. Figure 3a–c shows the mean eye-blink artifact component (the artifact region) of $\bar{a}$ obtained by the SSA method for window lengths M=22,32 and 64 and the parameter f=4,6,8,10 and 12 Hz. We have noticed from Figure 3a–c that the performance of SSA with f=4 Hz is low for different window lengths M=22,32 and 64. However, the performance of SSA with M=32 and 64 is stable for f=6,8,10 and 12 Hz, as evident from Figure 3d. The RRMSE curves were plotted with respect to the mean ground truth eye-blink artifact a. Based on the results in Figure 3d, the parameters of the SSA method, *f* and the window length *M* are set to 8 Hz and 64, respectively, to obtain better performance. For the proposed denoising methodology, Morlet wavelet transform has been used to represent the eye-blink artifact obtained by SSA into its time–frequency feature matrix, which is then given as input to the *k*-means clustering algorithm. In order to map the eye-blink artifact component into its time–frequency representation, we compute the wavelet coefficients in the range of 1 to 12 Hz with an increment of 0.25 Hz. This results in a feature matrix of size 45×2500. Such representation maps each sample of the eye-blink artifact estimated by SSA into a high-dimensional feature vector of size 45×1. It was clear from Figure 1 that the number of components (${\tilde{a}}^{1}$, ${\tilde{a}}^{2},$$\ldots ,{\tilde{a}}^{C}$) constructed using *k*-means information also increased when the number of clusters (C) increases. As the eye-blink artifact is a strong component, setting the number of clusters to 2 displayed better performance on short EEG epochs. Hence, we set the number of clusters to 2 for the proposed method. Based on the recommendations in [42], the parameters of the *k*-means+SSA method, the window length and thresholds $T_{h}$ and $T_{SSA}$ are set to 125, 1.4 and 0.01, respectively. In the case of SSA+ANC, we identified better performance with window length 40. Whereas in the case of the VME-DWT method, the α parameter is set to 1000 and the other parameters are fixed as in [29].

### 4.3. Results with Synthetic EEG Signals

**Figure 4:**
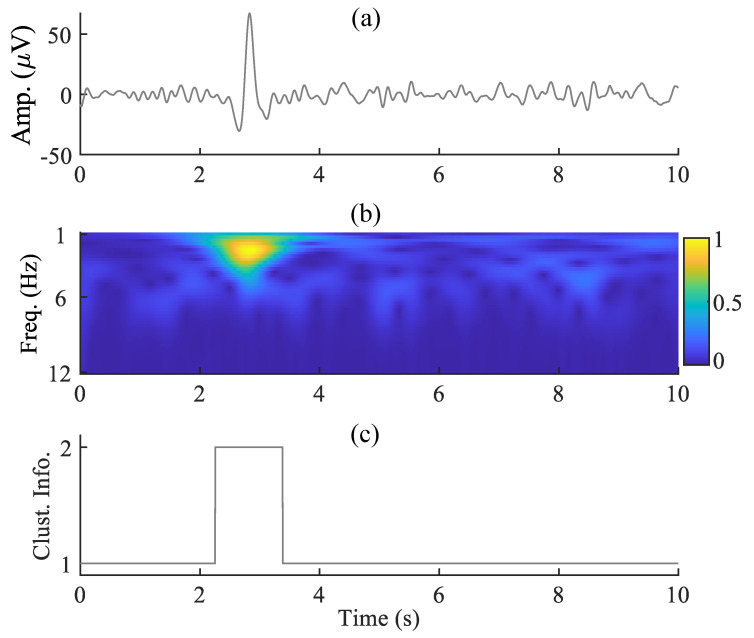
(**a**) The extracted eye-blink artifact ($\bar{a}$) by SSA, (**b**) its time–frequency representation using CWT (normalized plot) and (**c**) clustering information.

**Figure 5:**
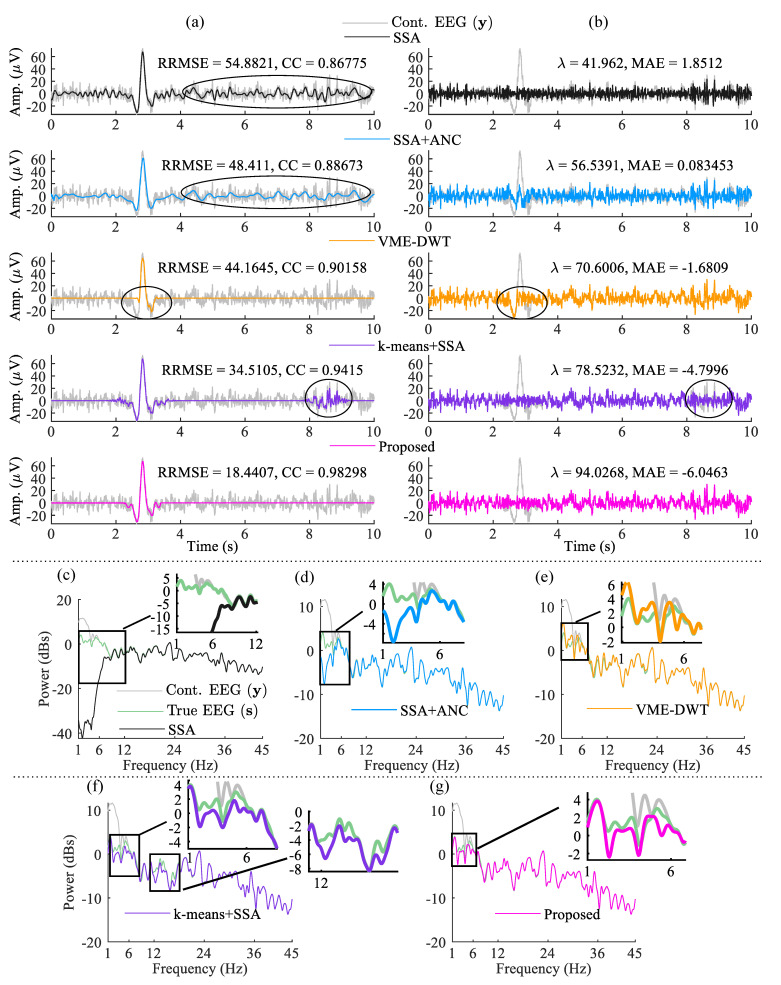
(**a**) The estimated eye-blink artifact ($\hat{a}$), (**b**) the corrected EEG signals ($\hat{s}$) using all methods, for the artifact mixing constant p=0.5. (**c**–**g**) the power spectrums of the true EEG (s), the contaminated EEG (y), and the corrected EEG signals of all methods.

The time–frequency representation of the extracted eye-blink artifact $\bar{a}$, in Figure 4a obtained by SSA, is shown in Figure 4b. As the eye-blink component is a strong component in $\bar{a}$, also evident from Figure 4a, the feature vectors of the time–frequency matrix (Figure 4b) between 2.5 and 3.5 s are significantly different. It is clear from the clustering information, shown in Figure 4c that all of the feature vectors (the columns of time-frequency map) corresponding to the eye-blink artifact region belong to cluster 2. The features vectors that correspond to the non-artifact region belong to cluster 1. By computing (12), we have obtained two signals ${\tilde{a}}^{1}$ and ${\tilde{a}}^{2}$, (as C=2). We have computed the FD of these two components to identify the eye-blink artifact. As the eye-blink artifact is a low-frequency component, we expect its corresponding FD to be a low value. Finally, the denoised eye-blink component is identified based on their FD value. The estimated eye-blink artifact and the corrected EEG signals using the proposed and the existing methods are shown in Figure 5. Even though the SSA and SSA+ANC methods extracted the eye-blink artifact very well, they also extracted the low-frequency EEG information from the non-artifact regions, as shown in Figure 5a. Although VME-DWT does not alter the non-artifact regions, it removed the eye-blink artifact partially (see circled region), whereas the *k*-means+SSA method removes valuable EEG information (see the circled region in the fourth row). In contrast, it is also clear from Figure 5b that there is no loss of EEG information with the proposed method. The RRMSE, the CC, the artifact reduction ratio (λ) and MAE values shown in Figure 5 also reveal the superiority of the proposed method over the existing methods. We also computed the power spectrums of the true EEG, the contaminated EEG and the corrected EEG signals to observe any spectral changes in the EEG signal after the artifact removal. Figure 5c–g shows the superposition plots of the true EEG, contaminated EEG and the corrected EEG signals using all methods. It can be observed from the power spectrum plots of the true and corrected EEG signals that the proposed method almost preserves the low-frequency information of the EEG signal as compared with the existing methods.

**Figure 6:**
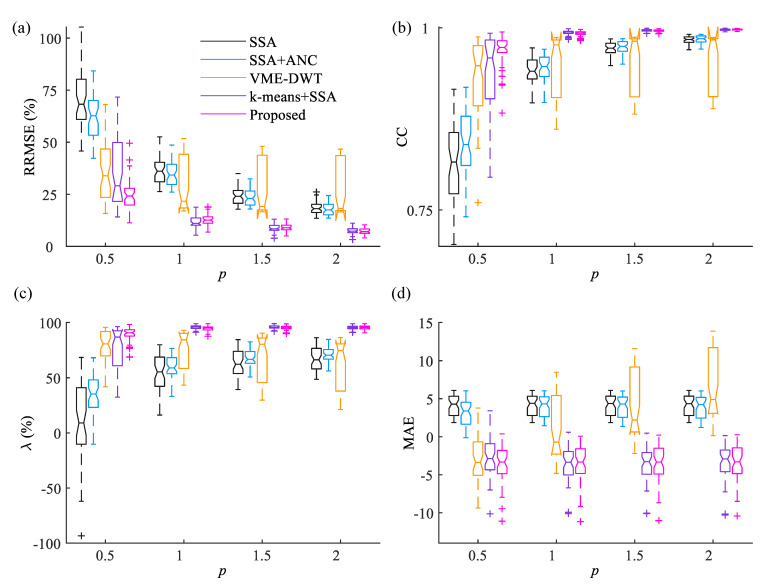
Performance of the existing and the proposed methods in terms of (**a**) RRMSE, (**b**) CC, (**c**) artifact removal ratio (λ) and (**d**) MAE (in log scale) with respect to the artifact mixing constant *p*. (The artifact mixing constant *p* is ∝1/SNR) of the signal.

We have applied the proposed method to remove the eye-blink artifacts from 50 synthetically contaminated EEG signals. Figure 6 shows the RRMSE, the CC, the artifact reduction ratio (λ) and the MAE plots obtained by the application of the existing and the proposed techniques over 50 EEG records for different artifact mixing constants (p). As discussed earlier, the artifact mixing constant *p* alters the SNR of the EEG signal. When p>1, the SNR of the EEG signal is low, whereas the SNR of the EEG signal is high for p<1. Removing the eye-blink artifact is a challenging task when its contribution in the contaminated EEG signal is low (i.e., p<1). The relation between *p* and SNR of the EEG signal is inversely proportional. The RRMSE and the CC values are computed with respect to the ground truth eye-blink artifacts. Whereas, the artifact reduction ratio (λ) and MAE values are computed with respect to the ground-truth EEG signals. It is clear from Figure 6a–d that in all conditions, the mean RRMSE, the CC, artifact reduction ratio and MAE values of the proposed method show better performance over SSA, SSA+ANC and VME-DWT methods. Although the VME-DWT showed comparative performance with the proposed method (see MAE plot) for p<1, its performance is poor for p≥1. Furthermore, the performance of the proposed method is better as compared to *k*-means+SSA for p<1 condition. Although the performance of the *k*-means+SSA method is comparable with the proposed method for p≥1, its performance is not stable due to the threshold parameters $T_{h}$ and $T_{SSA}$.

### 4.4. Results with Real EEG Signals

To evaluate the performance of the proposed method on the real EEG signals from the Fatigue EEG dataset (Fatigue EEG DB), we have segmented 50 EEG epochs of length 10 s from ten subjects’ lengthy EEG records [46,47]. Note that the data are re-sampled to 250 from 1000 Hz. Similarly, from the EEG Motor Movement/Imagery Database (EEG-MMI DB), an EEG epoch of 10 s from the lengthy EEG signal (eyes open task) obtained from 65 subjects is segmented [48,49]. The sampling frequency of this dataset is 160 Hz. For both datasets, the segmentation of the EEG epoch is performed such that at least one eye-blink artifact component is present in the segmented EEG epoch. From these two datasets, we have constructed in total 105 EEG epochs of length 10 s and evaluated the performance of the proposed and existing methods. In fact, for real EEG signals there will be no ground-truth EEG to evaluate the performance. Hence, we manually indicated the non-artifact and artifact intervals of each record and computed RRMSE and CC values.

**Figure 7:**
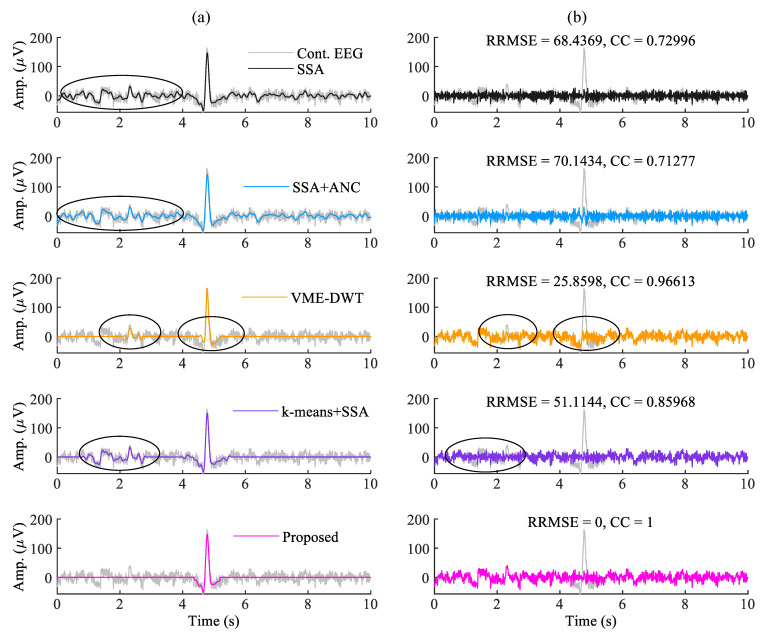
(**a**) The estimated eye-blink artifact ($\hat{a}$) and (**b**) the corrected EEG signals ($\hat{s}$) from the contaminated EEG signal (y) using the existing and the proposed methods.

The estimated eye-blink artifact and the corrected EEG signals (Fatigue EEG data) with all the methods are shown in Figure 7a,b. From Figure 7a, we can see that the low-frequency components are still present in eye-blink artifacts obtained by the SSA and SSA+ANC methods (see the non-artifact region between 1–4 s). As a result, low-frequency EEG information is removed from the corrected EEG signal obtained by the SSA and SSA+ANC, as shown in Figure 7b, whereas the VME-DWT method partially removed the eye-blink artifact and altered the non-artifact region in the time interval 2–3 s. The *k*-means+SSA method also altered the non-artifact region of the EEG signals in time interval 1–4 s (as indicated by circles in Figure 7b). The corrected EEG signal obtained by the *k*-means+SSA method and the contaminated EEG signals do not match in the non-artifact region (see 1–4 s in Figure 7b time interval). However, the corrected EEG signal obtained by the proposed method perfectly matches with the non-artifact region of the contaminated EEG signal, as shown in Figure 7b. The RRMSE and CC values shows the superiority of the proposed method over the existing methods.

As we do not have ground truth EEG signals for real EEG datasets, it is difficult to assess the performance of the proposed and existing methods in the frequency domain (power spectrum). However, the manually identified non-artifact and artifact regions of the EEG epoch are used to evaluate the performance of all methods in-terms of RRMSE and CC values. Table 1 shows the RRMSE and CC (mean ± standard deviation) values of the proposed method. Moreover, two binary classifier performance measures, such as precision and accuracy are also computed to evaluate the performance. Table 2 shows the mean precision and accuracy values of VME-DWT, *k*-means+SSA and proposed methods. It is also evident from Table 1 and Table 2 that the proposed method shows superior performance over the existing methods.

## 5. Discussion

Even though the SSA and SSA+ANC methods extract the eye-blink artifact component efficiently, they also alter the low-frequency component of the EEG signal in the non-artifact region (from Figure 5a and Figure 7a). However, subtracting the estimated eye-blink artifact directly from the contaminated EEG signal will also remove the low-frequency components (0.5–12 Hz) of the EEG signal. This can be a cause of concern in applications such as driver fatigue detection, where the spectral energy of low-frequency EEG components is used to detect the fatigue level [50]. The use of low-frequency EEG components to detect hand movements of subjects with spinal cord injury has been studied in [51,52]. In a recent study, it is found that the low-frequency EEG oscillations could be used as a biomarker of stroke injury and recovery [53]. Moreover, eye-blink component features (the frequency, amplitude and phase) are also used in applications such as control of hand exoskeleton for the paralyzed hand [54,55,56]. Therefore, in order to preserve these important low-frequency components at the pre-processing step, we combined SSA with CWT and *k*-means algorithms. The results show that the proposed method preserves these components while removing the eye-blink artifact. As the eye-blink artifact is a high amplitude component in the EEG signal (particularly in pre-frontal EEG channels), the proposed method has exploited this inherent feature to remove the eye-blink artifact without altering the original EEG components. Although the VME-DWT method does not alter the non-artifact intervals of EEG, it failed to remove the eye-blink artifact completely. Even though the *k*-means+SSA method displayed comparable performance as compared to the proposed method for a few EEG records, for cases where the eye-blink artifact is stronger, the proposed method fared well in overall performance. In this present study, we have only considered pre-frontal EEG channel signals. However, it can be expected from the results that the performance of the proposed method will be degraded further when the amplitude of the eye-blink artifact that is mixed in the EEG signal is low and this will be our topic of future research. For example, the eye-blink artifact contribution is low on fronto-central EEG channels $FC_{x}$.

## 6. Conclusions

In this paper, we combined SSA with CWT and the *k*-means algorithms to preserve the low-frequency EEG information in the artifact removal process. As the eye-blink artifact appears as a slow-time varying and strong component in the contaminated EEG signal, the proposed method exploited this feature to remove eye-blink artifacts from a single-channel EEG signal. The proposed method is evaluated on one synthetic and two real EEG datasets, and results show superior performance over existing techniques. Results also show the advantage of integrating SSA with CWT and *k*-means for eye-blink artifact removal from single-channel EEG signal. Since the present study considered the artifact removal from pre-frontal channel EEG signals, with the integration of available artifact detection algorithms, the proposed method could be employed for online applications where the pre-frontal EEG channel is used. Results show that the proposed method was successful in removing the eye blink artifact without the loss of original EEG information. Although the classification problem using the proposed method was not studied in the paper, we foresee that the proposed method will offer good performance in the final application. 

## Figures and Tables

**Table 1:** RRMSE and CC (μ±σ) comparison between the non-artifact interval of contaminated and corrected EEG signals.

Measures and Methods	Fatigue EEG DB		EEG-MMI DB	
	RRMSE	CC	RRMSE	CC
SSA	63.6077 ± 11.6133	0.7576 ± 0.1068	71.4361 ± 11.9799	0.6831 ± 0.1106
SSA+ANC	61.4721 ± 9.3272	0.7815 ± 0.0764	61.5249 ± 11.2345	0.7775 ± 0.0862
VME-DWT	6.7885 ± 13.3722	0.9885 ± 0.0283	5.9036 ± 10.9759	0.9922 ± 0.0164
*k*-means+SSA	16.3888 ± 16.4607	0.9713 ± 0.0598	16.0701 ± 13.7886	0.9770 ± 0.0361
Proposed	**4.9198 ± 7.4213**	**0.9960 ± 0.0139**	**2.9976 ± 7.3030**	**0.9969 ± 0.0104**

**Table 2:** Comparison of precision and accuracy (μ±σ) of the proposed method with existing methods for eye-blink detection on two real EEG datasets.

Measures and Methods	Fatigue EEG DB		EEG-MMI DB	
	Precision (%)	Accuracy (%)	Precision (%)	Accuracy (%)
VME-DWT	80.0445 ± 14.9771	93.8336 ± 5.0842	72.0040 ± 14.1527	92.8067 ± 4.9993
*k*-means-DWT	55.5252 ± 12.4375	82.7320 ± 8.7630	57.5738 ± 11.3917	86.8750 ± 7.8568
Proposed	**96.1604 ± 4.3639**	**94.2760 ± 6.3941**	**98.8142 ± 3.4201**	**95.4538 ± 2.6401**

## Data Availability

The EEG data and the MATLAB codes employed in this article will be made available by the authors upon request.
